# Emergence of the New KPC-49 Variant Conferring an ESBL Phenotype with Resistance to Ceftazidime-Avibactam in the ST131-H30R1 *Escherichia coli* High-Risk Clone

**DOI:** 10.3390/pathogens10010067

**Published:** 2021-01-14

**Authors:** Marta Hernández-García, Javier Sánchez-López, Laura Martínez-García, Federico Becerra-Aparicio, María Isabel Morosini, Patricia Ruiz-Garbajosa, Rafael Cantón

**Affiliations:** 1 Servicio de Microbiología, Hospital Universitario Ramón y Cajal and Instituto Ramón y Cajal de Investigación Sanitaria (IRYCIS), 28034 Madrid, Spain; martahernandez1986@gmail.com (M.H.-G.); jsl1991@hotmail.es (J.S.-L.); laurimartinezgarcia@gmail.com (L.M.-G.); fedebecerraaparicio@gmail.com (F.B.-A.); mariaisabel.morosini@salud.madrid.org (M.I.M.); rafael.canton@salud.madrid.org (R.C.); 2 Red Española de Investigación en Patología Infecciosa (REIPI), 28029 Madrid, Spain

**Keywords:** ST131-H30R1-*E. coli* high-risk clone, ceftazidime-avibactam susceptibility, *bla*_KPC-49_, whole genome sequencing

## Abstract

We report the emergence of an isolate belonging to the sequence type (ST)131-*Escherichia coli* high-risk clone with ceftazidime-avibactam resistance recovered from a patient with bacteremia in 2019. Antimicrobial susceptibility was determined and whole genome sequencing (Illumina-NovaSeq6000) and cloning experiments were performed to investigate its resistance phenotype. A KPC-3-producing *E. coli* isolate susceptible to ceftazidime-avibactam (MIC = 0.5/4 mg/L) and with non-wild type MIC of meropenem (8 mg/L) was detected in a blood culture performed at hospital admission. Following 10-days of standard ceftazidime-avibactam dose treatment, a second KPC-producing *E. coli* isolate with a phenotype resembling an extended-spectrum β-lactamase (ESBL) producer (meropenem 0.5 mg/L, piperacillin-tazobactam 16/8 mg/L) but resistant to ceftazidime-avibactam (16/4 mg/L) was recovered. Both *E. coli* isolates belonged to ST131, serotype O25:H4 and sublineage H30R1. Genomics analysis showed a *core* genome of 5,203,887 base pair with an evolutionary distance of 6 single nucleotide polymorphisms. A high content of resistance and virulence genes was detected in both isolates. The novel KPC-49 variant, an Arg-163-Ser mutant of *bla*_KPC-3_, was detected in the isolate with resistance to ceftazidime-avibactam. Cloning experiments revealed that *bla*_KPC-49_ gene increases ceftazidime-avibactam MIC and decreases carbapenem MICs when using a porin deficient *Klebsiella pneumoniae* strain as a host. Both *bla*_KPC-3_ and *bla*_KPC-49_ genes were located on the transposon Tn*4401*a as a part of an IncF [F1:A2:B20] plasmid. The emergence of novel *bla*_KPC_ genes conferring decreased susceptibility to ceftazidime-avibactam and resembling ESBL production in the epidemic ST131-H30R1-*E. coli* high-risk clone presents a new challenge in clinical practice.

## 1. Introduction

Global expansion of *Escherichia coli* sequence type 131 (ST131) among multidrug-resistant (MDR) *Enterobacterales* strains is a cause of great concern in Public Health. The ST131-*E. coli* clonal group associated with extended-spectrum-β-lactamase (ESBLs) production is the most frequent lineage among extraintestinal pathogenic *E. coli* (ExPEC) isolates [1,2]. The ST131 clone is part of the phylogenetic group B2 and predominantly corresponds to the O25b:H4 serotype [3]. The rapid and successful dissemination of the ST131 high-risk clone has been mainly attributed to the sublineage H30, defined by the presence of the specific fimbrial adhesin allele, *fimH30* [4]. Within the H30 sublineage, two fluoroquinolone resistant clades have been widely identified causing human infections: clade 1 or H30R1 and clade 2 or H30Rx [4,5]. The ST131-H30R1-*E. coli* subclone has been additionally related to an extensive virulence profile [1,2] and with the production of ESBL enzymes, particularly CTX-M-27 (sublineage C1-M27) [6].

In Spain, KPC (*Klebsiella pneumoniae* carbapenemase) production among ST131-*E. coli* isolates has been scarcely reported [7]. KPC enzymes hydrolyze efficiently almost all β-lactam antibiotics, and older β-lactamase inhibitors such as clavulanic acid are ineffective to prevent β-lactam degradation. Mobilization and diffusion of *bla*_KPC_ genes have been mostly linked to a conserved 10-kb Tn3-based transposon (Tn4401) and a wide variety of conjugative plasmids that frequently harbor resistance genes against other antimicrobial groups [8]. Infections caused by these MDR KPC-producing Enterobacterales isolates are usually difficult to treat and are frequently associated with high mortality and morbidity rates [9,10]. In February 2015, the U.S. Food and Drug Administration (FDA) approved the ceftazidime-avibactam combination as an alternative to carbapenems in patients with complicated intra-abdominal and urinary tract infections caused by MDR Gram-negative bacteria isolates [11]. Since then, several studies have demonstrated the in vivo efficacy of ceftazidime-avibactam in the treatment of infections caused by KPC-producers [12,13,14]. Moreover, a recent study has supported the presumptive use of ceftazidime-avibactam against carbapenem resistance *E. coli* isolates, including members of the ST131 lineage [15]. Nevertheless, the recent description of *K. pneumoniae* high-risk clones producing new KPC variants conferring resistance to ceftazidime-avibactam is a cause of great concern in the clinical setting [16,17,18].

The aim of this work was to describe the emergence of the novel KPC-49 variant conferring ceftazidime-avibactam resistance in an ST131-H30R1-*E. coli* isolate, with an ESBL resembled phenotype, recovered from an infected patient during the ceftazidime-avibactam treatment.

## 2. Results

### 2.1. Case Report

**Figure 1:**
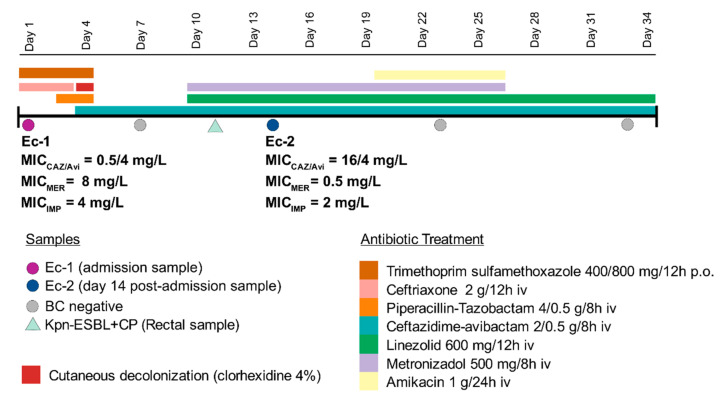
Timeline of events and antibiotic treatments received during the admission. CAZ/Avi = ceftazidime-avibactam; MER = meropenem; IMP = imipenem; BC = blood culture; Kpn = *K. pneumoniae*; p.o. = oral dosage; iv = intravenous.

In April 2019, a 40–50 range age woman with a biliary cholangiocarcinoma was admitted at the Ramón y Cajal University Hospital (Madrid, Spain) with sepsis symptoms. Timeline of events during the hospital admission and antibiotic treatments received are represented in Figure 1. An *E. coli* isolate with an MDR phenotype consistent with carbapenemase production (Ec-1) was recovered at admission from a blood culture. Ec-1 showed non wild-type susceptibility to imipenem (MIC = 4 mg/L) and meropenem (MIC = 8 mg/L) and co-resistance to other antimicrobial groups, except ceftazidime-avibactam (MIC = 0.5/4 mg/L), tigecycline (MIC ≤ 0.5 mg/L), amikacin (MIC ≤ 8 mg/L), colistin (MIC ≤ 1 mg/L), and fosfomycin (MIC ≤ 32 mg/L) (Table 1). The eazyplex ^®^SuperBug CRE system demonstrated the presence of a *bla*_KPC_ gene, and following the regional guidelines [19], the patient was placed under contact precautions in a single room.

At admission, the patient was treated with ceftriaxone (2 g/12 h iv) and trimethoprim sulfamethoxazole (400/800 mg/12 h po) during 3 and 4 days, respectively. Piperacillin-tazobactam (4/0.5 g/8 h iv) was also added between days 3 and 4. Treatment with ceftazidime-avibactam (2/0.5 g/8 h iv) was started on day 4 and was discontinued at discharge (after 30 days). On day 4, following the protocol of contact precautions, the patient’s skin was washed with a 4% soapy chlorhexidine antiseptic gel. On day 7, a sterile blood culture was recovered, but due to the recurrent fever, the antibiotic regimen was modified adding metronidazole (500 mg/8 h iv) and linezolid (600 mg/12 h iv) over ceftazidime-avibactam from day 10 to 27 (17 days) and to day 34 (24 days), respectively. On hospital day 12, a *K. pneumoniae* isolate with a phenotype compatible with KPC carbapenemase production was detected in a rectal culture. Unfortunately, this isolate was not preserved for subsequent analysis.

On day 14 post-admission, 10 days after the ceftazidime-avibactam treatment initiation, a second KPC-producing *E. coli* isolate (Ec-2) was recovered in a blood sample. This isolate (Ec-2) exhibited a phenotype compatible with an ESBL producer and showed an MIC reduction of 1-fold dilution to imipenem (MIC = 2 mg/L) and 4-fold dilutions to meropenem (MIC = 0.5 mg/L) with piperacillin-tazobactam in the susceptible, increased exposure category (I) (MIC = 16/4 mg/L). On the contrary, an increase of 5-fold dilutions to ceftazidime-avibactam MIC (16/4 mg/L) was observed compared to Ec-1 isolate. Moreover, Ec-2 remained susceptible to tigecycline (MIC ≤ 0.5 mg/L), amikacin (MIC ≤ 8 mg/L), colistin (MIC ≤ 1 mg/L) and fosfomycin (MIC ≤ 32 mg/L) (Table 1). The eazyplex ^®^SuperBug CRE system also confirmed the presence of a *bla*_KPC_ in this isolate. On day 20, amikacin (500 mg/24 h iv) was administered until day 27 and blood cultures collected between days 23 and 33 were negative. Patient’s fever remitted and patient was discharged 34 days post-admission (Figure 1).

### 2.2. Genome Characteristics and E. coli Typing

Assembly of both Ec-1 and Ec-2 strains revealed an approximate genome size of 5.2 Mb with a G+C content of 50.7%. Information about genomes characteristics is summarized in Table S1. The functional classification of both annotated genomes using the KEGG2 database showed a majority of genes linked to metabolism (44%), followed by genetic information processing (28%) and signaling and cellular processes (25%). Phylogenetic analysis of Ec-1 and Ec-2 isolates showed a *core* genome of 5,203,887 base pair (bp) with six single nucleotide polymorphisms (SNPs) (evolutionary distance of 1.15 SNPs/Mb) and one of them was the R163S mutation in *bla*_KPC-3_ (Table S2) designated as *bla*_KPC-49_ (accession number MN619655). Ec-1 and Ec-2 isolates were identified as ST131 by the Achtman scheme. Both ST131 isolates belonged to the serotype O25:H4 and were assigned to phylogroup B2. Moreover, both strains carried the *fimH30* allele and were identified as subclone H30R1 (clade 1).

### 2.3. Resistance and Virulence Profile

Ec-1 and Ec-2 isolates contained the same antibiotic resistance genes conferring resistance to β-lactams (*bla*_KPC-*like*_, *bla*_OXA-9_, *bla*_TEM-1_, *bla_EC-6_*), aminoglycosides (*aac*3-IId, *aad*A5, *aph*(3″)-Ib and *aph*(6)-Id), macrolides (*mph*A-*mrx*), sulfonamides (*sul*1, *sul*2), tetracycline (*tet*A), and trimethoprim (*dfr*A17). Genes encoding *bla*_CTX-M_ or other carbapenemases were not detected. Ec-2 isolate carried the novel variant *bla*_KPC-49_, differing from *bla*_KPC-3_ by a single nucleotide substitution (nt 487, C-A) resulting in the arginine-for-serine substitution at amino acid position 163 (R163S). Genes encoding multidrug resistance efflux pumps (*acrAB*-*TolC*, *H-NS*, *emr* and *mdt*) and acid resistance systems (*gad*XW) were also identified in both *E. coli* isolates. Moreover, fluoroquinolone resistance mutations in quinolone resistance-determining region (QRDR), including *gyrA* (S83L, D87N), *parC* (S80I, E84V), and *parE* (I529L), were found in both Ec-1 and Ec-2 isolates.

An identical virulence factor composition was detected in both ST131-*E. coli* isolates: aslA, chuA, chuS-Y, entB-F, entS, fdeC, fepA-D, fepG, fes, fimC-I, fyuA, irp1-2, iucA-D, iutA, kpsD, ompA, papB, papX, sat, senB, yagV/ecpE, yagW/ecpD, yagX/ecpC, yagY/ecpB, yagZ/ecpA, ybtA, ybtE, ybtP, ybtQ, ybtS, ybtT, ybtU, ybtX, ykgK/ecpR.

### 2.4. bla_KPC_ Genetic Environment

KPC-3 and the novel KPC-49 variant were located on an IncFII plasmid also harboring FIA/FIB replicons. In silico pMLST typing confirmed that this IncF-FIA-FIB plasmid belonged to the F1:A2:B20 sequence type. Both *bla*_KPC-3_ and *bla*_KPC-49_ genes were found as part of the composite transposon Tn4*401* (Tn*3*) designated as “isoform *a*” variant.

### 2.5. Resistance Phenotype Conferred by bla_KPC-49_ Gene

pKPC-3 and pKPC-49 recombinant plasmids were successfully obtained by cloning. Subsequently, pKPC-3 or pKPC-49 were transformed into the isogenic *E. coli* DH5-α strain. Additionally, with the purpose of investigating the resistance phenotype conferred by KPC-49 enzyme in comparison to KPC-3 in a genetic background more similar to the most common *K. pneumoniae* clinical strains, pKPC-3 and pKPC-49 plasmids were also transformed into the porin deficient SHV-5-producing *K. pneumoniae* CSUB10R (ΔompK35; ΔompK36; ΔompK37) strain. Changes in the MIC values of ceftazidime-avibactam and carbapenem antibiotics were non observed between pKPC-3- and pKPC-49 *E. coli* DH5-α transformants using microdilution and MIC gradient strips (Table 1). Nevertheless, an increased ceftazidime-avibactam MIC (≥3.5-fold) and a reduction of the MIC values for carbapenems, particularly imipenem (≥3-fold), were detected by gradient strips in the pKPC-49-*K. pneumoniae*-CSUB10R transformant over the corresponding pKPC-3-*K. pneumoniae-*CSUB10R (Table 1).

## 3. Discussion

To the best of our knowledge, we report for the first time an ST131-H30R1-*E. coli* strain producing a new KPC variant, the R163S mutant of *bla*_KPC-3_, conferring decreased susceptibility to ceftazidime-avibactam and designated as KPC-49. This ST131-*E. coli* strain was detected in a blood sample recovered from a patient during the antibiotic treatment with ceftazidime-avibactam (day 10). Typing characterization by WGS identified this ST131 strain as the H30R1 subclone, serotype O25:H4. The ST131-H30R1-*E. coli* subclone has been recently described in Europe as an emerging MDR pathogen involved in rectal colonization and associated with CTX-M-27 production (C1-M27 subclade) [6].

Association of ST131 strains with the production of KPC enzymes has been scarcely described and the few reports are in countries with a high endemicity level of KPC-producing *K. pneumoniae* [20,21]. For instance, a high incidence of the high-risk clone ST307-KPC-3-producing *K. pneumoniae* has been recently reported in both colonized and infected patients in our institution during the last two years (unpublished data). The persistence of certain *K. pneumoniae* clones, such as ST307, in the patient microbiome plays a crucial role in the cross-species transfer of carbapenemase genes [22,23]. Interestingly, a KPC-producing-*K. pneumoniae* isolate was also detected in a rectal sample from our patient during the admission. So that, we cannot rule out that the presence of this isolate in the patient microbiome could have facilitated the horizontal acquisition of *bla*_KPC-3_ gene by the ST131-*E. coli*.

Overall, the dissemination of *bla*_KPC_ among different Enterobacterales species has been mainly related to the transposable element Tn*4401*a which is often carried on different conjugative plasmids [24]. In fact, cross-species transfer of IncF *bla*_KPC-3_-encoding plasmids from *K. pneumoniae* to *E. coli* have been previously described in infected patients [25]. On the other hand, IncFII plasmids with FIA/FIB replicons have also been detected among ST131-*E. coli* isolates, mostly linked to the *bla*_ESBLs_ genes dissemination. IncF (F1:A2:B20) plasmids have been usually associated with the H30R1 clade and IncF (F2:A1:B-) plasmids with the H30Rx clade [26,27]. Coincidentally, we found the ST131-H30R1 subclone harboring an IncF (F2:A1:B20) plasmid but encoding *bla*_KPC-3_ and the novel *bla*_KPC-49_ variant, not an ESBL-encoding gene. Furthermore, in contrast with previous studies [28], we found in both ST131-*E. coli* isolates a complete Tn*4401a* transposon, identical to that described in *K. pneumoniae* strains during the last decade. In fact, Tn*4401a* has been previously identified in our hospital as the genetic platform involved in the mobilization and diffusion of the *bla*_KPC-3_ gene in *K. pneumoniae* [24]. On the other hand, although *bla*_CTX-M_ genes has been largely found inserted in both IncF-type plasmids and chromosomal locations in ST131-*E. coli* isolates [28], CTX-M-encoding genes were not detected in our isolates.

Coinciding with the reported literature, a high number of virulence and resistance genes was identified in both KPC-3- and KPC-49-ST131-H30R1-*E. coli* isolates [1,5]. The ST131-*E. coli* isolate recovered at admission showed a multidrug resistance profile, leaving few therapeutics options, as ceftazidime-avibactam, to treat the blood infection.

Although the in vivo clinical efficacy of ceftazidime-avibactam has been widely demonstrated [12,13], several studies have recently reported in *K. pneumoniae* epidemic clones the emergence of new ceftazidime-avibactam resistant KPC enzymes derived from point mutations in *bla*_KPC-2_ and *bla*_KPC-3_ genes, frequently after the antibiotic exposure [16,17,18]. In all cases, in vitro meropenem susceptibility was fully or partially restored due to these *bla*_KPC_ mutations resulting in KPC-producing *K. pneumoniae* isolates with a phenotype resembling that of ESBL producers [16,17,18]. According to these data, the impact of ceftazidime-avibactam resistance could be minimized due to the lower carbapenem MICs. However, some studies have shown that after the discontinuation of ceftazidime-avibactam treatment, carbapenem resistant phenotype can be restored in *K. pneumoniae* isolates that still display ceftazidime-avibactam resistance [18]. In our study, a higher susceptibility to meropenem, imipenem, and piperacillin-tazobactam was observed in the KPC-49-ST131 strain coinciding with the increased ceftazidime-avibactam MIC. In concordance with previous reports, this resistance phenotype resembled that of an ESBL producer [16,17,18]. Importantly, our region is considered an endemic area of ESBL-producing *E. coli* isolates [29] and the emergence of the ST131-*E. coli* producing KPC carbapenemases but with an ESBL resistance profile could lead to under reporting of infections by KPC producers with potential carbapenemase activity.

According to our results, this resistance phenotype could be consequence of the R163S mutation in *bla*_KPC-3_. It is noteworthy that the *bla*_KPC-49_ was more clearly validated as a ceftazidime-avibactam resistance determinant in the porin-deficient strain *K. pneumoniae* CSUB10R. Previous studies have also demonstrated that mutated or non-functional OmpK35 and Ompk36 porins slightly contribute to increase ceftazidime-avibactam MICs in KPC-3-producing *K. pneumoniae* isolates [30,31]. It should also be noted that the *K. pneumoniae* CSUB10R strain shares a similar genetic background to the MDR-*K. pneumoniae* clinical strains that usually circulate in the hospital setting causing severe and difficult-to-treat infections. We believe that the potential contribution of the ST131-*E. coli* clone in the dissemination of novel *bla*_KPC_ genes conferring ceftazidime-avibactam resistance, such as *bla*_KPC-49_, in epidemic *K. pneumoniae* clinical strains should be a cause of great concern for public health.

## 4. Material and Methods

### 4.1. Bacterial Isolates, Susceptibility Testing, and Patient’s Data

Bacterial identification was carried out using MALDI-TOF-MS (Bruker-Daltonics, Bremen, Germany). Minimum inhibitory concentrations (MICs) were determined by standard broth microdilution. Ceftazidime-avibactam susceptibility was additionally tested using gradient strips (Liofilchem, Roseto degli Abruzzi, Italy). We defined susceptible isolates as those categorized as S (susceptible, standard dose regimen) and I (susceptible, increased exposure) according to EUCAST-2019 criteria (http://www.eucast.org/clinical_breakpoints/). Clinical chart review was performed after local ethical committee approval (Ref. 293/19).

### 4.2. Resistance Genes Characterization

Carbapenemase genes were detected initially by the eazyplex^®^-Superbug-CRE system (Amplex-Biosystems GmbH, Giessen, Germany) and later confirmed by PCR and Sanger sequencing [32].

### 4.3. DNA Extraction, Whole Genome Sequencing, and Bioinformatics Analysis

Total DNA extraction from 2 mL exponential growth cultures was performed by the Chemagic DNA Bacterial External Lysis Kit (PerkinElmer Inc., Waltham, MA, USA). Whole genome sequencing was carried out by the Illumina NovaSeq 6000 platform (OGC, Oxford, UK) with 2 × 150 pb paired-end reads. Quality control and filtering of sequences were performed using FastQC v.0.11.8 (https://www.bioinformatics.babraham.ac.uk/projects/fastqc/) and Prinseq-lite-0.20.3 (http://prinseq.sourceforge.net/) tools, respectively. Preprocessing short-reads were assembled *de novo* using SPAdes v3.11.1 [33] and assembly evaluation was performed by QUAST v5.0.2 (http://quast.bioinf.spbau.ru/). The draft genomes were annotated by Prokka v.1.13.3 [34] and the functional categorization of predicted protein sequences were also classified using the KEGG Automatic Annotation Server and the KEGG2 database (http://www.genome.jp/kegg/kaa). Antimicrobial resistance and virulence genes were identified using Abricate v0.8.11 with the ResFinder, ARG-ANNOT, CARD and VFDB databases (threshold, 98% identity; 90% coverage). MLST v2.16.1 (https://github.com/tseemann/mlst) and the Achtman MLST scheme (http://mlst.warwick.ac.uk/mlst/dbs/Ecoli) were used in the in silico MLST assignment. Phylogroups, serotypes and fimH types were determined using ClermonTyping [35], SerotypeFinder and FimTyper (https://cge.cbs.dtu.dk/services/) tools, respectively. H30Rx sublineage was identified based on the G723A point mutation in *ybbW* using Blastn (http://blast.ncbi.nlm.nih.gov/Blast.cgi) [36]. Chromosomal point mutations (*gyrA*, *parC* and *parE* genes) were identified using PointFinder software (https://cge.cbs.dtu.dk/services/). Variant calling was performed by Snippy v4.3.2 program. BWA-MEM v0.7.17 and Samtools v1.9 softwares were also used to confirm the presence of mutations between isolates. The genetic environment and the mobile genetic elements implicated in the dissemination of the *bla*_KPC_ gene were characterized using PlasmidFinder and Plasmidspades v3.11.1 [37].

### 4.4. Validation of bla_KPC-49_ Variant

*bla*_KPC-3_ and *bla*_KPC-49_ genes were amplified by PCR using primers KPC-F (5′-AGGAATATCGTTGATGTCACT-3′) and KPC-R (5′-CTTACTGCCCGTTGACGCC-3′) and cloned into the pCR-Blunt II-TOPO vector following the manufacturer’s instructions (Zero Blunt TOPO PCR cloning kit; Invitrogen, Cergy-Pontoise, France). The recombinant plasmids pKPC-3 and pKPC-49 were transformed by heat shock into competent *E. coli* cells (NEB 5-alpha competent *E. coli*; New England BioLabs Inc., Ipswich, MA, USA) and then selected on Luria broth agar medium supplemented with ampicillin (30 mg/L), kanamycin (50 mg/L) and IPTG (isopropyl-β-D-thiogalactopyranoside)-Xgal (5-bromo-4-chloro-3-indolyl-β-D-galactopyranoside) (80 mg/L). Both pKPC-3 and pKPC-49 plasmids were also transferred by electroporation into the porin-deficient SHV-5-producing *K. pneumoniae* CSUB10R strain (ΔompK35; ΔompK36; ΔompK37) [38] and then selected in Luria broth agar medium supplemented with imipenem (4 mg/L). Successful transfer of *bla*_KPC_ genes was confirmed by PCR and Sanger sequencing. MIC values in KPC-3 and KPC-49 transformants were also measured by broth microdilution. Additionally, most of the β-lactam antibiotics including ceftazidime-avibactam were tested in triplicate using MIC test strips.

### 4.5. Sequence Data

The *bla*_KPC-49_ gene and the complete genomes of both ST131-H30R1-*E. coli* isolates were deposited at DDBJ/ENA/GenBank under accession numbers MN619655, WIRF00000000 and WIRG00000000, respectively.

## 5. Conclusions

In the present work we report the in vivo emergence of a novel KPC variant (KPC-49) conferring a phenotype resembling that of ESBL producers in the ST131-*E. coli* high-risk clone during the treatment with ceftazidime-avibactam. The global expansion of the ST131-H30-*E. coli* high-risk clone in both community and hospital settings together with the successful acquisition of IncF-type plasmids harboring *bla*_KPC_ genes that function as ESBL genes but also conferring resistance to new β-lactam and β-lactamase inhibitor combinations such as ceftazidime-avibactam, poses a new challenge to the patient management and the containment programs design to avoid the spread of MDR pathogens.

## Figures and Tables

**Table 1:** Antimicrobial susceptibility results by broth microdilution and gradient strips for KPC-producing *E. coli* isolates (Ec-1 and Ec-2), KPC-producing *E. coli* transformants (pKPC-3-TM and pKPC-49-TM), the isogenic *E. coli* DH5α strain, KPC-producing *K. pneumoniae* transformants (pKPC-3-TM and pKPC-49-TM), and the SHV-5-producing *K. pneumoniae* CSUB10R strain (ΔompK35; ΔompK36; ΔompK37).

	Broth Microdilution (mg/L) (Interpretation)									Gradient Strips (mg/L) (Interpretation)				
Antimicrobials	Ec-1	Ec-2	*E. coli* pKPC-3-TM	*E. coli* pKPC-49-TM	*E. coli* DH5α	*K. pneumoniae* pKPC-3-TM	*K. pneumoniae* pKPC-49-TM	*K. pneumoniae* CSUB10R	*E. coli* pKPC-3-TM	*E. coli* pKPC-49-TM	*E. coli* DH5α	*K. pneumoniae* pKPC-3-TM	*K. pneumoniae* pKPC-49-TM	*K. pneumoniae* CSUB10R
Piperacillin-tazobactam	>64/4 (R)	16/4 (I)	≤4/4 (S)	≤4/4 (S)	≤4/4 (S)	>64/4 (R)	>64/4 (R)	>64/4 (R)	-	-	-	-	-	-
Ceftazidime	>32 (R)	>32 (R)	1 (S)	2 (I)	≤0.25 (S)	>32 (R)	>32 (R)	>32 (R)	0.25 (S)	1 (S)	0.19 (S)	>256 (R)	>256 (R)	>256 (R)
Cefotaxime	>32 (R)	>32 (R)	≤0.25 (S)	≤0.25 (S)	≤0.25 (S)	>32 (R)	>32 (R)	>32 (R)	0.032 (S)	0.047 (S)	0.032 (S)	>256 (R)	>256 (R)	>256 (R)
Cefepime	>64 (R)	64 (R)	≤0.125 (S)	0.25 (S)	≤0.125 (S)	>64 (R)	>64 (R)	64 (R)	0.047 (S)	0.047 (S)	0.023 (S)	>256 (R)	>256 (R)	32 (R)
Aztreonam	>32 (R)	>32 (R)	4 (I)	≤0.5 (S)	≤0.5 (S)	>32 (R)	>32 (R)	>32 (R)	-	-	-	-	-	-
Ceftazidime-avibactam	0.5/4 (S)	16/4 (R)	≤0.125/4 (S)	≤0.125/4 (S)	≤0.125/4 (S)	4/4 (S)	4/4 (S)	2/4 (S)	0.047/4 (S)	0.094/4 (S)	0.094/4 (S)	2/4 (S)	12/4 (R)	2/4 (S)
Imipenem	4 (I)	2 (S)	0.5 (S)	≤0.25 (S)	≤0.25 (S)	>16 (R)	>16 (R)	2 (S)	0.25 (S)	0.25 (S)	0.38 (S)	>32 (R)	4 (I)	0.75 (S)
Meropenem	8 (I)	0.5 (S)	≤0.125 (S)	≤0.125 (S)	≤0.125 (S)	16 (R)	8 (I)	2 (S)	0.023 (S)	0.023 (S)	0.032 (S)	6 (I)	2 (S)	2 (S)
Gentamicin	>8 (R)	>8 (R)	≤8 (S)	≤8 (S)	≤8 (S)	≤8 (S)	≤8 (S)	≤8 (S)	-	-	-	-	-	-
Amikacin	≤8 (S)	≤8 (S)	≤8 (S)	≤8 (S)	≤8 (S)	≤8 (S)	≤8 (S)	≤8 (S)	-	-	-	-	-	-
Tobramycin	>8 (R)	>8 (R)	≤8 (S)	≤8 (S)	≤8 (S)	≤8 (S)	≤8 (S)	≤8 (S)	-	-	-	-	-	-
Ciprofloxacin	>2 (R)	>2 (R)	≤2 (S)	≤2 (S)	≤2 (S)	>2 (R)	>2 (R)	>2 (R)	-	-	-	-	-	-
Tigecycline	≤0.5 (S)	≤0.5 (S)	≤0.5 (S)	≤0.5 (S)	≤0.5 (S)	≤0.5 (S)	≤0.5 (S)	≤0.5 (S)	-	-	-	-	-	-
Colistin	≤2 (S)	≤2 (S)	≤2 (S)	≤2 (S)	≤2 (S)	≤2 (S)	≤2 (S)	≤2 (S)	-	-	-	-	-	-
Fosfomycin	≤32 (S)	≤32 (S)	≤32 (S)	≤32 (S)	≤32 (S)	>32 (S)	>32 (S)	>32 (S)	-	-	-	-	-	-

## Supplementary Materials

The following are available online at https://www.mdpi.com/2076-0817/10/1/67/s1, Table S1: Genomic characteristics of KPC-producing *E. coli* isolates; Table S2: SNPs detected in the *core* genome of both KPC-producing *E. coli* isolates.
