# Supplementary material for: Emergence of the New KPC-49 Variant Conferring an ESBL Phenotype with Resistance to Ceftazidime-Avibactam in the ST131-H30R1 Escherichia coli High-Risk Clone
Source: Pathogens. 2021 Jan 14;10(1):67. doi: 10.3390/pathogens10010067 (PMC7828710; doi:10.3390/pathogens10010067)
Supplement: Supplementary file 1 [file pathogens-10-00067-s001.pdf]

Table S1. Genomic characteristics of KPC-producing *E. coli* isolates.

	**Ec-1**	**Ec-2**
Total length (pb)	5236588	5243162
Contigs	172	228
Contigs (>= 500pb)	79	89
N50	165103	165107
L50	11	10
GC (%)	50.74	50.73
CDS	4986	4992
tRNA	74	71
tmRNA	1	1

Table S2. SNPs detected in the *core* genome of both KPC-producing *E. coli* isolates

**Effect in Ec-2**	**Gene**	**Product**
Synonymous_variant c.1296G>T p.Val432Val	napA	Periplasmic nitrate reductase
Missense_variant c.246C>A p.His82Gln		ISNCY family transposase ISRor2
Synonymous_variant c.54C>T p.Asp18Asp	fliM	Flagellar motor switch protein FliM
Intergenic_region n.10310C>G		tRNA-Met
Missense_variant c.1299A>T p.Leu433Phe	fdrA_3	Protein FdrA
**Missense_variant c.487C>A p.Arg163Ser**	***bla*$_{KPC-3}$**	**Carbapenem-hydrolyzing beta-lactamase KPC**
